# Two Tier Slicing Resource Allocation Algorithm Based on Deep Reinforcement Learning and Joint Bidding in Wireless Access Networks

**DOI:** 10.3390/s22093495

**Published:** 2022-05-04

**Authors:** Geng Chen, Xu Zhang, Fei Shen, Qingtian Zeng

**Affiliations:** 1 College of Electronic and Information Engineering, Shandong University of Science and Technology, Qingdao 266590, China; sxxyzx@163.com (X.Z.); qtzeng@sdust.edu.cn (Q.Z.); 2 Shanghai Institute of Microsystem and Information Technology, Chinese Academy of Sciences, Shanghai 200050, China; fei.shen@mail.sim.ac.cn

**Keywords:** network slicing (NS), resource allocation, deep reinforcement learning, bidding

## Abstract

Network slicing (NS) is an emerging technology in recent years, which enables network operators to slice network resources (e.g., bandwidth, power, spectrum, etc.) in different types of slices, so that it can adapt to different application scenarios of 5 g network: enhanced mobile broadband (eMBB), massive machine-type communications (mMTC) and ultra-reliable and low-latency communications (URLLC). In order to allocate these sliced network resources more effectively to users with different needs, it is important that manage the allocation of network resources. Actually, in the practical network resource allocation problem, the resources of the base station (BS) are limited and the demand of each user for mobile services is different. To better deal with the resource allocation problem, more effective methods and algorithms have emerged in recent years, such as the bidding method, deep learning (DL) algorithm, ant colony algorithm (AG), and wolf colony algorithm (WPA). This paper proposes a two tier slicing resource allocation algorithm based on Deep Reinforcement Learning (DRL) and joint bidding in wireless access networks. The wireless virtual technology divides mobile operators into infrastructure providers (InPs) and mobile virtual network operators (MVNOs). This paper considers a single base station, multi-user shared aggregated bandwidth radio access network scenario and joins the MVNOs to fully utilize base station resources, and divides the resource allocation process into two tiers. The algorithm proposed in this paper takes into account both the utilization of base station (BS) resources and the service demand of mobile users (MUs). In the upper tier, each MVNO is treated as an agent and uses a combination of bidding and Deep Q network (DQN) allows the MVNO to get more resources from the base station. In the lower tier allocation process, each MVNO distributes the received resources to the users who are connected to it, which also uses the Dueling DQN method for iterative learning to find the optimal solution to the problem. The results show that in the upper tier, the total system utility function and revenue obtained by the proposed algorithm are about 5.4% higher than double DQN and about 2.6% higher than Dueling DQN; In the lower tier, the user service quality obtained by using the proposed algorithm is more stable, the system utility function and Se are about 0.5–2.7% higher than DQN and Double DQN, but the convergence is faster.

## 1. Introduction

With the advent of the 5G era, the demand and application of mobile traffic and wireless networks have increased dramatically. This huge demand has driven the convergence of multiple traditional and emerged communications technologies to form the 5G mobile communications system. 5G mobile communication systems employ new technologies and new network architectures that enable them to go beyond traditional communications and meet the needs of different types of devices and users [1]. As defined by the International Telecommunication Union (ITU), 5G mobile communication system considers three common scenarios with specific service requirements: eMBB, mMTC, and URLLC [2,3,4]. EMBB is a scenario with ultra-high transmission data rate and mobility guarantee under wide coverage, it helps ensure consistent user experience [5], mMTC mainly deals with the scalable connectivity to a massive number of MTC devices and sensors with diverse quality of service (Qos) requirements [6]. URLLC is mainly faced with application scenarios with latency-sensitive and high-reliability requirements for delay and reliability [7]. With NS techniques, a 5G network can be divided into multiple logical networks on a separate physical network for services with different requirements. The origin of NS technology can be traced back to the infrastructure as a service (IAAs) cloud computing model [8]. With this model, different tenants can share the compute resources, network resources, and storage resources, thus creating different isolated and fully functional virtual networks on a common infrastructure. NS manages physical and virtual resources based on emerging technologies such as software defined network (SDN) and network function virtualization (NFV), so that they can be provided to specific services [9,10,11], enabling 5G networks to provide different types of services to customers with different needs [12,13], and the network slice assumes a static resource pool for each slice to ensure the performance isolation between different types of slices [13,14]. Wireless NS technology divides the existing mobile network operators (MNOs) into two functionally distinct entities: InPs and MVNOs. Each InPs has a certain physical wireless network, including physical infrastructure and physical resources [15]. MVNOs divide the physical wireless network under their own InPs to get each MVNOs exclusive virtual wireless network and rent the physical resources owned by the InPs to provide specific services or meet specific service demands for their own MUs. In this way, the sharing of physical resources can effectively reduce operating expenses and enable more flexible network operations. Additionally, resources allocated to their own MUs must meet strict service level agreements (SLAs). And in resource allocation, the actual needs of users are often determined by the way they request [16].

In the actual network resource allocation problem, users’ statuses are often fluctuating, and operators are not aware of parts of users’ information such as channel conditions. Therefore, how MVNO optimally allocates resources for users is a key issue of this research. To solve these optimization problems, recently, some studies have proposed new approaches such as game theory approaches [17], linear programming approaches [18], etc. In this paper, we mainly adopt the approach of using DRL combined with bidding to solve such optimization problems. For this optimization problem of resource allocation, many algorithms have been proposed in the literature in recent years. In this paper, some algorithms (some heuristic algorithms) are considered when selecting algorithms. Due to their own characteristics, they can not guarantee to obtain the global optimal solution when solving the problem that will produce a huge state space, with poor convergence and high complexity, and the setting of parameters during simulation will have a great impact on the experimental results. The optimization problem considered in this paper has many unknown parameter variables (such as channel state and user information), which produces a huge state space. Fortunately, the emerging DRL is considered a promising technique to solve this complex control problem. Therefore, this paper attempts to use DRL joint bidding to solve the proposed optimization problem. It is found that DRL is very suitable for the scenarios and optimization problems to be considered. Actions with a high matching degree can be set in the environment, and states and rewards can be mapped to DRL, which can be trained to get better strategies. The emerging DRL is considered a promising technique to solve this complex control problem [19]. Ref. [20] use a DRL algorithm to solve this optimization problem and get good results. This new intelligent algorithm can learn knowledge that is not available through traditional methods by big data training, and uses trial-and-error search methods to interact dynamically with the environment in real time, which enables unprecedented automation and optimization of resource allocation [21]. It is also a good approach to treat the resource allocation problem as a resource game issue, which needs to consider the dynamic competing behaviors of users to maximize the overall satisfaction of users [22].

At present, a lot of research work has been done based on these two methods. Ref. [23] used an allocation strategy of orthogonal and multiplexed subchannels to ensure the isolation of inter-slice and solved the problem of minimizing system power in the bidirectional transmission link. Ref. [24] proposed a new auction-based shared resource and revenue optimization model. Ref. [25] proposed a stochastic game model to solve the dynamic resource allocation problem of multi-user virtual enterprise networks and proposed a blind approximate based great likelihood estimation algorithm to solve the model, thus overcoming the cost of information exchange and computation, but the model does not consider user-specific demands. Ref. [26] mathematically analyzes the joint optimization of access control and bandwidth allocation for multiple BS and multiple NS scenarios. However, the solution is based on the assumption that different users have the same fixed demand rate, which is unlikely to be found in practice. Ref. [27] proposed an LSTM-based prediction scheme, and use a power allocation algorithm based on DRL to solve this problem. But in practical scenarios, different types of user demands need to be considered when solving the network resource allocation problem. Ref. [28] proposed an optimization framework based on a resource pricing strategy to maximize resource efficiency and customer profit by studying the relationship between profit maximization and resource efficiency. Ref. [29] proposed an AC priority algorithm to meet the high demand and high priority slice to improve the overall resource demand satisfaction rate, ref. [30] used game theory to analyze the relationship between InPs and users to optimize the allocation problem and solve the communication problem during peak hours. Ref. [31] used communication games and learning mechanisms to solve the distributed problem of wireless NS resources, but without considering the deployment of users with different types of demand. Ref. [32] proposed an online resource management for inter-slice genetic slicing policy optimizer, but it ignores the relationship between the required resources on the different types of slices and the SLA. Ref. [33] proposed a novel channel information absent Q-learning (CIAQ) algorithm to speed up the training, but this algorithm is only an auxiliary method for solving the resources allocation problem. Reference [34] considered the problem of allocating different types of resources (bandwidth, cache, backhaul capacity) to network service tenants based on user demand and proposed a mathematical solution, but when the simulation parameters are increased proportionally, the optimization problem will become difficult to solve. Ref. [35] uses a DRL method to control the energy of the UAV scene. Ref. [36] proposed a DNAF-based DQL merging method that improves the convergence speed of the algorithm. Ref. [37] proposed an HA-DRL algorithm, that uses heuristic functions to optimize the exploration of action space.

The bidding methods and the DRL methods have been proposed in the above literature to solve the resource allocation problems of BS to different users. But some methods do not consider that users are with different specific needs, and the resource allocation to users has the problem of poor service quality or waste of resources for a proportion of users, moreover, some solutions simply consider the service satisfaction rate of users and ignore the total bandwidth of BS. Some solutions simply consider the user’s service satisfaction rate and ignore the total bandwidth utilization rate of the BS, which also results in the waste of wireless network resources. To solve the challenges and problems mentioned above, this paper proposes a two-tier resource allocation model considering both the BS resource utilization and user service satisfaction rate. In fact, this paper decomposes a single objective optimization problem into two-level sub-objective optimization problems, and creatively uses DRL to solve the two-level resource allocation optimization problem considering the inconsistency between the upper and lower value spaces. The upper tier model is for MVNOs to request resources from the BS by bidding, and this paper uses a combination of bidding and Dueling DQN to solve the optimization problem of this upper tier model. Likewise, the lower tier model is for MVNOs to allocate the resources which are received from the BS to its contained users and set the service satisfaction rate of the users, the same as the upper tier, the lower tier model is optimized using Dueling DQN. The main contributions of this paper are as follows.

(1) First, a two tier resource allocation problem in wireless NS is proposed. The upper tier MVNOs will submit bid prices to the InP for wireless resources. The InP will further allocate physical resources to the MVNOs based on the bid values of the MVNOs. Each MVNO will then use the wireless resources allocated by the network to serve its mobile subscribers.

(2) Second, the algorithm based on Dueling DQN and joint bidding is used to solve the upper tier resource allocation optimization problem. In this paper, the utility of each MVNO is obtained by calculating the downlink transmission rate of the user after obtaining the bandwidth, and the utility function of the whole system is denoted as the weighted sum of the upper tier benefits and the lower layer utility function. This ensures that the BS resources are allocated to the maximum extent possible to meet the service demand of the users more efficiently.

(3) Third, this paper shows the process of mathematical analysis of the proposed two-tier model and algorithm with its corresponding parameters for problem solving, and shows how bidding can be used in conjunction with Dueling DQN with the corresponding parameters. The penalty function is proposed to prevent the MVNO from overbidding, and the evaluation function to represent the revenue of the MVNO. This paper considers a radio access network scenario with multiple users sharing aggregated bandwidth under a single BS, where users are randomly located within the range of the BS and have different service demands, the BS does not have direct access to the channel information and service demand information of the users, and each MVNO manages the users in a sub-region. In future research work, it can take into consideration changes in user location and changes in service demand, in order to get closer to the actual communication scenario.

The rest of the paper consists of the following: Section 2 presents the two-tier model proposed in this paper with its mathematical analysis process. Section 3 presents the solution algorithm and the relevant mathematical background, and details the process of corresponding parameters when using the Dueling DQN and DQN algorithm in the two-tier model. The simulation process and results are given in Section 4, and a comparative analysis is performed. Section 5 concludes the paper and gives an expectation.

## 2. System Model and Problem Formulation

**Figure 1:**
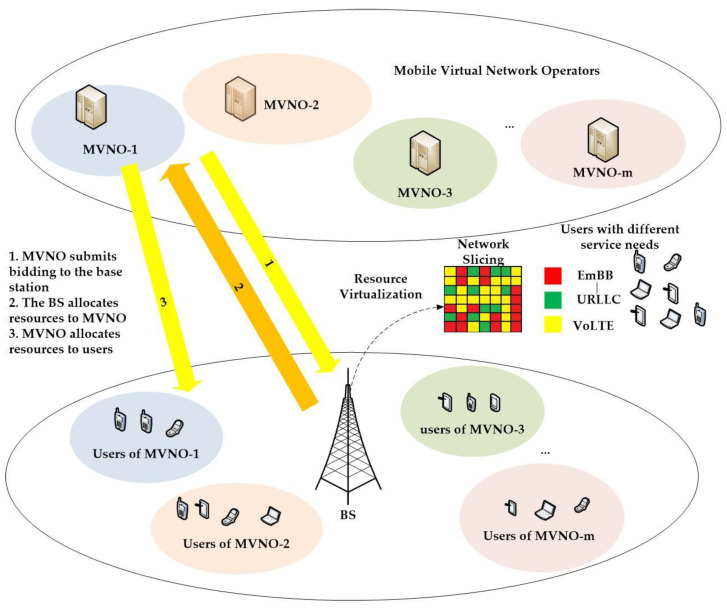
Downlink transmissions scenario and the relationship between BS, MVNOs, and users.

In this part, we consider a downlink scenario with a single BS, as shown in Figure 1. This single BS is divided into a physical BS and a set of MVNOs, $M=\left\{M_{1},\;M_{2},\ldots ,M_{m}\right\}$, each MNVO has j users $U_{m}=\left\{u_{j}^{m},u_{j}^{m},\ldots ,u_{j}^{m}\right\}$ connected, and each MVNOS provides specific mobile services to its connected users. This BS has resources (shared aggregated bandwidth) C. Each MVNO is required to bid resources to the BS according to the demands of the connected users and allocate the resources received from the BS to its connected users. In this paper, the SLA satisfaction rate (SSR) is used to represent the quality of experience (QoE) of the users. The core problem of this paper is how to schedule among the MVNOs and satisfy the demands of the connected users and maximize the total profit of MVNO. Moreover, the resources of this BS are virtualized and sliced to meet the demands of the users. The resource allocation problem after NS is divided into two tiers.

### 2.1. Upper Tier Model

In the upper tier model, based on the number and the QoS requirements of users it connects, each MVNO has to decide the required wireless bandwidth and estimate a bid value to submit to InP. The InP will allocate a proportion of its resources (bandwidth) to each MVNO based on the MVNO’s bid value, which means that the InP will allocate the largest part of bandwidth to the MVNO which submits the highest bid [6]. The resources allocated by the BS to the mth MVNO are denoted as $c_{m}$, and the resources allocated by the mth MVNO to the users are denoted as $c_{j}^{m}$, and each MVNO will count the minimum rate demand $v_{j,0}^{m}$, and the maximum rate demand $v_{j,1}^{m}$ of its linked users and estimate from these demands.

Each MVNO gets the minimum rate demand $v_{j,0}^{m}$, the maximum rate demand $v_{j,1}^{m}$ and the bid value $b_{m}$ of each MVNO.

(1) $$\sum_{j=0}^{J}v_{j,0}^{m}<b_{m}<\sum_{j=0}^{J}v_{j,1}^{m}$$

(2) $$c_{m}=\frac{b_{m}}{\sum_{m=1}^{M}b_{m}}C,\;\forall m\in M$$

(3) $$y_{m}(c_{m}\left(b\right))=v_{m}\left(c_{m}\left(b\right)\right)-q_{m}b_{m}$$

The BS are allocated resources $c_{m}\left(b\right)$ to MVNOs in proportion to their bids. To prevent MVNOs from excessively increasing their bids, an evaluation function $y_{m}\left(c_{m}\left(b\right)\right)$ is established, and $q_{m}$ as a penalty function which will reduce the profit of MVNOs if they excessively increase their bids, and α is represented by function (5).

(4) $$q_{m}=\frac{1}{\alpha }v_{m}{}^{\prime }\left(1-\frac{c_{m}\left(b\right)}{C}\right)$$

(5) $$\alpha =\frac{\sum_{m=1}^{M}b_{m}}{R}$$

The optimization problem of the upper model is to maximize the weighted sum of the benefits and utility of all MVNOs, i.e.,

(6) $$\mathrm{maxF}=\sum_{m\in M}f_{m}+\omega *\sum_{m\in M}y_{m}\left(c_{m}\left(b\right)\right)$$

(7) $$S.\;t.\;\;c_{m}\left(b\right)\cap c_{n}\left(b\right)=0$$

(8) $$\sum_{m\in M}c_{m}\leq C$$

(9) $$\sum_{j=1}^{J}c_{j}^{m}\;\leq c_{m}$$

Constraint: constraint (6) ensures the segregation of the resources allocated between different MVNOs. Since the bandwidth of the BS is limited, constraint (7) ensures that the bandwidth allocated to all MVNOs does not exceed the total bandwidth of the BS, and constraint (8) means that the sum of the bandwidth allocated by each MVNO to its connected users cannot be greater than the bandwidth allocated to itself from the BS. The problem of each MVNO getting resources by bidding can also be solved by DQN, the exact process of which will be mentioned later.

### 2.2. Lower Tier Model

The MVNO is allocated by the resources received from the InP by the upper tier to the connected users, and the main task in the lower tier model is to find a suitable bandwidth allocation scheme to maximize the utility function of each MVNO, labeled $f_{m}$, and the utility function $f_{m}$ can be expressed as a weighted sum of $SE_{m}$ and $SSR_{u_{j}^{m}}$. The computation of $SE_{m}$ and $SSR_{u_{j}^{m}}$ is described in the following section.

From Shannon’s formula, it can be calculated that $v_{j}^{m}$, $v_{j}^{m}$ denotes the downlink rate from the BS to the jth user $u_{j}^{m}$ which is linked to mth MVNO.

(10) $$v_{j}^{m}=c_{j}^{m}log\left(1+SNR_{u_{j}^{m}}\right)$$

(11) $$v_{m}=\sum_{j=1}^{J}v_{j}^{m}$$

$u_{j}^{m}$ denotes the jth user of the mth MVNO, and $SNR_{u_{j}^{m}}$ is the signal-to-noise ratio with the BS $u_{j}^{m}$.

(12) $$SNR_{u_{j}^{m}}=\frac{g_{u_{j}^{m}}P}{N_{0}c_{j}^{m}}$$

$g_{u_{j}^{m}}$ denotes the fading gain of the channel between the BS and $u_{j}^{m}$, P denotes the transmitted power, and $N_{0}$ denotes the one-sided noise spectral density.

(13) $$SE_{m}=\frac{\sum_{u_{j}^{m}\in U_{m}}\sum_{j\in J}v_{u_{j}^{m}}}{c_{m}}$$

$SSR_{u_{j}^{m}}$ denotes the SSR of the jth user connected by the mth MVNO

(14) $$SSR_{u_{j}^{m}}=\frac{\sum_{q_{j}^{m}\in Q_{j}^{m}}\alpha_{q_{j}^{m}}}{\sum Q_{j}^{m}}$$

In this paper, the SSR is expressed as the ratio of the number of valid packets successfully accepted by the user to the total number of packets sent by the MVNO. $q_{j}^{m}$ denotes the packet successfully accepted by the user $u_{j}^{m}$, and binary $\alpha_{q_{j}^{m}}$ denotes whether the accepted $q_{j}^{m}$ packet is valid, when $v_{u_{j}^{m}}>\bar{v_{u_{j}^{m}}}$, $\alpha_{q_{j}^{m}}=1$, otherwise $\alpha_{q_{j}^{m}}=$0. $\bar{v_{u_{j}^{m}}}$ is the downlink transmission rate that is preset in advance for the user $u_{j}^{m}$ according to the SLA.

(15) $$\max\;\;\;\;f_{m}=\max(\rho SE_{m}+\sum_{j\in J}\varphi_{j}\;SSR_{u_{j}^{m}})$$

The optimization objective of the lower-tier model is to maximize the total utility function $f_{m}$ for each MVNO, and $f_{m}$ can be expressed as a weighted sum of SE and SSR. ρ and $\varphi =\left\{\varphi_{1},\varphi_{2},\ldots ,\varphi_{s}\right\}$ denotes the important weights of SE and SSR, respectively.

Notably, this optimization process can be analyzed as a Markov decision process, but trying to solve (15) is difficult, and using traditional assignment or using the Q-learning algorithm does not yield a better solution quickly. Fortunately, DRL is useful for solving such problem, the process of mapping to the Dueling DQN algorithm will be mentioned later.

## 3. DRL-Based Joint Bidding Resource Allocation Algorithm

### 3.1. Deep Reinforcement Learning

DQN is a typical DRL algorithm, it is advantageous for solving high computational problems and decision problems. In DRL, there will be an agent to control the learning process. The intelligent agent attempts to generate a lot of new data through constant trial-and-error interaction with the environment, and then learns a set of policies based on this data that enables it to maximize the cumulative expected reward while finding the best action for a given state. We can model the agent’s interaction with the environment as a Markov decision process (S,A,R,P,γ).

The parameters are explained as follows: S is the state space containing the current state s and the new state $s^{\prime }$; A is the action space containing the current action a and the new action $a^{\prime }$; the policy π(·|s) determines how state s is mapped to the action; R is the reward function obtained by performing the action a under the state s according to the policy π(·|s); P(·|s,a) is the transfer probability and γ is a discount factor.

Additionally, the state value function $V^{\pi }\left(s\right)$ can be obtained according to π(·|s) under the state s.

(16) $$V^{\pi }\left(s\right)=E_{\pi ,P}\left[\sum_{t=0}^{\infty }\gamma^{t}R_{t}|S_{0}=s\right]$$

Similarly, the action value function $Q^{\pi }\left(s,a\right)$ obtained by executing the action a under the state s according to the policy π(·|s).

(17) $$Q^{\pi }\left(s,a\right)=E_{\pi ,P}\left[\sum_{t=0}^{\infty }\gamma^{t}R_{t}|S_{0}=s,A_{0}=a\right]$$

The process of interaction between the intelligent body and the environment is as follows: the agent gets an observation as a state s from the environment and inputs s to the neural network to get all $Q^{\pi }\left(s,a\right)$, then uses the ϵ-greedy strategy selects an action and makes a decision from $Q^{\pi }\left(s,a\right)$, and the environment will give a reward and the next observation based on this action. Finally, the agent is updated according to the reward given by the environment using Equation (17).

(18) $$Q^{*}\left(s,a\right)=Q\left(s,a\right)+\alpha \left(R+\gamma max_{a^{\prime }}Q\left(s^{\prime },a^{\prime }\right)-Q\left(s,a\right)\right)$$

DQN is based on DL with the addition of neural networks with parameters θ for parameter updating and action selection. The Q-value function network is updated in real time and the target Q-value function network is updated every certain number of iterations. Q(s,a;θ) denotes the value function with parameters θ, the optimal parameters θ will be obtained by minimizing the TD error squared according to Equation (18) to let $Q\left(s,a;\theta \right)=Q^{*}\left(s,a\right)$.

(19) $$\zeta^{2}={\left[r+\gamma max_{a^{\prime }\in A}Q\left(s^{\prime },a;\theta \right)-Q_{\theta }\left(s,a;\theta \right)\right]}^{2}$$

The target Q-value of the network of target Q-value functions is

(20) $$TargetQ=r+\gamma max_{a^{\prime }}Q\left(s^{\prime },a;\theta \right)$$

Also, the loss function defined in L(θ) DQN is

(21) $$L\left(\theta \right)=E\left[{\left(TargetQ-Q\left(s,a;\theta \right)\right)}^{2}\right]\;$$

While Dueling DQN improves on the network structure of DQN, Dueling DQN divides the Q value into two parts, one for the state value function, and one for the advantage function, denoted as:

(22) $$Q_{DuelingDQN}{}^{\pi }\left(s,a\right)=V^{\pi }\left(s\right)+A^{\pi }\left(s,a\right)$$

$V^{\pi }\left(s\right)$ is unconcerned with action a, and only one status value is returned, while $A^{\pi }\left(s,a\right)$ is related to action and state, $Q_{Dueling}{}^{\pi }\left(s,a\right)$ can be expressed in more detail as:

(23) $$Q_{Dueling}\left(s,a;\theta ,\alpha ,\beta \right)=V^{\pi }\left(s;\theta ,\alpha \right)+A^{\pi }\left(s,a;\theta ,\beta \right)$$

The parameters θ in the formula are shared by the two function networks, and α and β are their exclusive parameters. In order to increase the identification of the two functions, the dominant function is generally centralized, that is:

(24) $$Q_{Dueling}\left(s,a;\theta ,\alpha ,\beta \right)=V^{\pi }\left(s;\theta ,\alpha \right)+A^{\pi }\left(s,a;\theta ,\beta \right)-\frac{1}{A}\sum_{a^{\prime }\in A}A\left(s,a^{\prime };\theta ,\beta \right)$$

### 3.2. Two Tier Slicing Resource Allocation Algorithm Based on Dueling DQN and Joint Bidding

In actual communication, due to various factors, the channel information and service demands of users are private. In order to better meet the user demand and to maximize the utilization rate of physical resources in the BS, MVNO is added between the BS and users. The MVNOs collect the users’ demand information and channel status, then bid and obtain resources from the BS, finally allocate resources to users connected to it. This paper mapped the above problem to a Markov decision process, uses the framework of bidding for the upper tier model in the allocation process, and uses the DRL for both two tiers to solve the optimization problem, get the optimal solution by iterative training.

Algorithm 1 uses the DQN joint with the framework of bidding to solve the optimization problem for the upper tier model. After initializing the bidding pool *B*, the parameters in the neural net within the DQN (such as ($Q,\;\theta ,\;\alpha ,\;\beta ,\;\hat{Q},$ and *N*). In the simulation, each MVNO obtains the bidding range to establish a bidding pool *B*, the total maximum and minimum demand resources of the users of the MVNO are first estimated, which is represented by the maximum and minimum value of the sum of the expected rates (set by SLA) of all users connected to it. It is used to indicate the maximum rate requirement of each MVNO if the service requirement of each user is the service type with the maximum rate. After converting the rate requirement to the maximum and minimum bid value according to a specific ratio, the bid pool *B* can be established. The upper tier uses the bid pool *B* as the action space, and the maximum lower tier action corresponding to each upper tier action is found in the lower tier and stored in table *A*.
**Algorithm 1** DQN and Joint Bidding Algorithm for Upper Tier Bandwidth Allocation 1:**Initialize** the Bidding pool *B* of MVNO and corresponding lower tier action selection table *A*;2:**Initialize** the action-value function Q, target action-value function $\hat{Q}$ the replay memory *D* to capacity *N*3:Each MVNO m∈M estimates the maximum total needed rate and minimum total needed rate of linked users, then create the Bidding pool *B*;4:**For** $b_{m}$ in *B*
**do**5: 
Find the lower tier optimal allocation action and store it in table *A*;6:**end for**7:Random choose an action $a_{t}$ i.e., bidding value $b_{m}\epsilon B$ and BS distributes $c_{m\;}$ to each MVNO according to (2);8:**Repeat**9: 
**For**
*t* = 1, to *T*, **do**10:  Calculate the ratio of the allocated bandwidth to its required minimum rate, and take it as the current state *S = s* of the last iteration;11:  For *m* = 1 to *M*, do12:   
Each MVNO m allocates optimal bandwidth $c_{j}^{m}$ to its users according to table *A*;13:   
Each MVNO m calculates the $v_{m}$ by (9) and (10);14:   
Each MVNO m calculates the penalty $q_{m}$ by (4); 15:   
Each MVNO m and calculates the profit $y_{m}$ by (3) and get the reward $r_{m}$;16:
  **End for**
17:  Calculate the total system utility F according to (5);18:  Calculate the total reward *r*;19:  Choose an action $a_{t}$ i.e., bidding value $b_{m}\epsilon B$ according to the policy of DQN; 20:  InP distributes $c_{m\;}$ to each MVNO according to (2); 21:  Get the state *S = s’* after the selection action of this iteration;22:  #Train DQN23:  The agent i.e., each MVNO inputs $\left(s,a,\;s^{\prime },r\right)$ into the DQN;24:  The agent stores transition $\left(s,a,\;s^{\prime },r\right)$ in *D*;25:  The agent sample random minibatch of transitions $\left(s\_,a\_,\;s^{\prime }\_,r\_\right)$ from *D*;26:  Set$$y\_=\{\begin{array}{c} r_{-}\\ r\_+\gamma max_{a^{*}}\hat{Q}\left(s^{\prime }\_,a^{*};\theta^{-}\right) \end{array}\begin{array}{c} \mathrm{if}\;\mathrm{episode}\;\mathrm{terminates}\;\mathrm{at}\;\mathrm{step}\;\_+1\\ \mathrm{otherwise} \end{array}$$
27:  The agent perform a gradient descent step on ${\left(y\_-Q\left(s\_,a\_;\theta \right)\right)}^{2}$ with respect to the network parameters *θ*;28:  Every steps reset $\hat{Q}=Q$;29:
 
**End for**
30:**Until** The predefined maximum number of iterations has been completed.

Before starting the iteration, an upper tier action needs to be randomly selected to generate the initial state. The components of the iteration process include: getting the current state s, selecting the action a according to the policy π(·|s) in the current state s and generating the state s_, calculating the utility function F, and calculating the reward r. At the beginning of each iteration, the current state s is available. In combination with the DQN algorithm, the actions in each iteration are selected according to the DQN policy, the ϵ-greedy policy, randomly selected an action or selected a better action according to $a_{t}=argmax_{a}Q\left(\varphi \left(s_{t}\right),\;a;\theta \right)$. The action $a=a_{t}$ of each iteration contains the bids of each MVNO in this iteration $a=\left\{b_{1},b_{2},\ldots ,b_{m}\right\}$. The InP receives the bids $b_{m}$ from MVNOs and divides the bandwidth resources proportionally to each MVNO bandwidth $c=\left\{c_{1\;},\;c{\;}_{2},\ldots ,c_{m}\right\}$ according to Equation (2). Each MVNO will allocate bandwidth $c_{m}$ to each user and count the rate $v_{m}$ sum of each user, each MVNO can get the ratio of the allocated bandwidth to its required minimum rate, and take it as the next state s_. The MVNO also constructs an action space when allocating bandwidth to users, and the optimal lower-tier action $a_{l}$ corresponding to each upper-tier action can be found based on table A. Then, the MVNO derives a discount function from Equation (4) and calculates the profit value $y_{m}$ in this iteration from Equation (3) based on the sum of $v_{m}$ and $q_{m}$. When all MVNOs in this iteration have performed the above actions, the total utility function F and the total reward r of the system in this iteration is counted.

Finally, the s, a, s_ and r generated by this iteration are input into the DQN and trained. In DQN, the agent stores the transition $\left(s,\;a,\;s^{\prime },\;r\right)$ of each iteration into the experience pool D, then takes a small random transition $\left(s\_,\;a\_,\;s^{\prime }\_,\;r\_\right)$ from the experience pool D for training the parameters of the Q-value net, finally updates the parameters of the target Q-value net by the loss function L(θ).

Algorithm 2 uses the Dueling DQN algorithm to solve the optimization problem of the lower-level model. As in Algorithm 1, the parameters ($Q,\theta ,\hat{Q}$, and *N*) in the Dueling DQN neural network are first initialized and each MVNO creates its lower tier action space $A_{l}$ after receiving the resources $c_{m}$ allocated from the BS. Before each iteration, each MVNO will randomly select an action $a\in A_{l}$ from its lower action space and execute it. The action a first divides its resources into resource blocks for three services, then allocates resources $c_{j}^{m}$ to users which are connected to it, then count the number of packets successfully received $q_{j}^{m}$ by the user and denote it as state s. Then start the iteration, the agent i.e., MVNO will get the current state s, and choose an action a according to the policy of the Dueling DQN policy, the ϵ-greedy policy, randomly selects an action or selected a better action according to $a_{t}=argmax_{a}Q\left(\varphi \left(s_{t}\right),\;a;\theta ,\alpha ,\beta \right)$, after the allocation process, MVNO counts the state $s^{\prime }$, utility function $f_{m}$ and reward r, finally, input the $\left(s,a,\;s^{\prime },r\right)$ into the Dueling DQN and train the neural network until the predefined maximum number of iterations has been completed.
**Algorithm 2** Dueling DQN Algorithm for Lower Tier Bandwidth Allocation1:**Initialize** the action-value function Q, target action-value function $\hat{Q}$ the replay memory *D* to capacity *N*2:Each MVNO receives a bandwidth $c_{m\;}$ from the BS;3:Each MVNO creates an action space $A_{l}$;4:**For***m* = 1 to *M*, **do**5: MVNO randomly chooses an action $a\in A_{l}$ and performs a;6: MVNO allocates the bandwidth $\;c_{j}^{m}$ to users which are connected with it;7: Calculate the $q_{j}^{m}$ as state s;8: **For**
*t* = 1, to *T*, **do**9:  The agent gets the current state s;10:  Choose an action $a\in A_{l}$ according to the policy of Dueling DQN; 11:  Calculate the total system utility $f_{m}$ according to (15);12:  Calculate the total reward;13:  The agent allocates the bandwidth to users and calculates the state after the selection action of this iteration as $s^{\prime }$;14:  #Train Dueling DQN15:  The agent i.e., each MVNO inputs $\left(s,a,\;s^{\prime },r\right)$ into the Dueling DQN;16:  The agent store transition $\left(s,a,\;s^{\prime },r\right)$ in *D*;17:  The agent sample random minibatch of transitions $\left(s\_,a\_,\;s^{\prime }\_,r\_\right)$ from *D*;18: Set $$y\_=\{\begin{array}{c} r_{-}\\ r\_+\gamma max_{a^{*}}\hat{Q}\left(s^{\prime }\_,a^{*};\theta^{-},\alpha ,\beta \right) \end{array}\begin{array}{c} \mathrm{if}\;\mathrm{episode}\;\mathrm{terminates}\;\mathrm{at}\;\mathrm{step}\;\_+1\\ \mathrm{otherwise} \end{array}$$
19:  The agent perform a gradient descent step on ${\left(y\_-Q\left(s\_,a\_;\theta ,\alpha ,\beta \right)\right)}^{2}$ with respect to the network parameters *θ*, α and β;20:  Every steps reset $\hat{Q}=Q$;21: **End for**
22:**End for**

## 4. Simulation Results and Discuss

Compared with the latest published literature in recent years, as Table 1, this paper considers the sliced bandwidth resources as a two tier resource allocation process, and ensures the service quality of users’ multiple service requirements. Through the simulation, we get good results by using the DRL joint bidding.

### 4.1. Simulation Parameters

In the scenario considered in this paper, the maximum aggregated bandwidth provided by a single BS is 10 MHz, and the minimum specification of the bandwidth resource block is set to $r_{block}$ = 0.2 MHz, three types of services (i.e., VoLTE, eMBB, and URLLC) and four MVNOs are provided to the subscribers, and 100 registered subscribers are randomly present within an approximate circle of 40 m radius around the BS. The transmission power of the users is 20 dBm, and the transmit power of the BS is 46 dBm. The noise spectral density of the channel is −174 dBm/Hz under the given channel model. The minimum rate constraint for VoLTE service is 51 kbs, the minimum rate constraint for eMBB service is 0.1 Gb/s, and the minimum rate constraint for URLLC service is 0.01 Gb/s. The detailed simulation parameters are shown in the following Table 2.

The simulation sets up 100 users randomly distributed in a single BS coverage area, and the users have three different service demand types (i.e., VoLTE, eMBB, and URLLC), and the service demand of each user is also random. An MVNO is set up to pre-allocate the BS resources between the BS and the users, and the users are connected to different MVNOs according to their locations. To demonstrate the feasibility and advantages of the proposed resource allocation algorithm, the following work is carried out in this paper.

Firstly, the proposed model based on bidding and a two tier Dueling DQN algorithm is simulated through the python platform and simulated with a Double DQN algorithm, DQN algorithm. and Q-Learning algorithm. After getting the data of the four algorithms plotted graphs and comparing, it is concluded that the algorithm proposed in this paper is feasible and has some advantages over the other three algorithms in this paper. The following is the curve and comparative analysis after plotting some data obtained from this simulation.

In the process of simulation for the training network parameters set the reward is calculated as:

The upper tier reward = 4 + (SE − 230) * 0.1 + (profit − 185) * 0.1 (if the Qoe of eMBB ≥ 0.975, the Qoe of Volte ≥ 0.98, the Qoe of URLLC ≥ 0.95, the SE ≥ 220 and the profit ≥ 185). In the preceding conditions, if SE is not satisfied, the reward = 4; if profit is larger than 170 but also not satisfied the conditions, the reward = (the Qoe of URLLC − 0.7) * 10; and if the Qoe of URLLC also not satisfied, reward = (the Qoe of URLLC − 0.7) * 6; if just satisfied the first condition, reward = 0, and if each condition is no satisfied, reward = −5.

The lower tier rewards are a bit simpler to set up and are part of the rewards that consist of the upper tier: reward = 4 + (SE − 280) * 0.1 (if the Qoe of eMBB ≥ 0.96, the Qoe of Volte ≥ 0.98, the Qoe of URLLC ≥ 0.95, the SE ≥ 280); reward = 4 (if SE not satisfied); reward = (the Qoe of URLLC − 0.7) * 10(if the Qoe of URLLC is not satisfied); reward = −5 (if each conditions is not satisfied).

In particular, in the upper model, we evaluated the method of joint bidding of Doble DQN and Dueling DQN, and compared it with the results of traditional DQN, Double DQN, Dueling DQN, and Q-learning. In the experiment, the learning rates of various algorithms are set to 0.01. And the importance weight of the optimization objective obtained by formula (6) and formula (15) is set to ρ=0.01, φ=[1,1,1], ω=0.1. The learning rate of the Dueling DQN network is set to 0.01, and the choice of Gama value was experimentally set to 0.95.

In the whole simulation process, 100 user locations are randomly distributed, with the BS location as the origin, and 4 MVNOs manage four areas, respectively, and collect their service demands. In this paper, as Table 3, the service types of the users connected by MVNO-0 include 11 eMBB services, 9 VoLTE services, and 7 URLLC services; the service types of the users connected by MVNO-1 include 11 eMBB services, 8 VoLTE services, and 7 URLLC services; the service types of the users connected by MVNO-2 include 8 eMBB services, 6 VoLTE services, and 13 URLLC services. 6 VoLTE services and 13 URLLC services; MVNO-3 connected users’ service types include 2 eMBB services, 8 VoLTE services, and 7 URLLC services.

### 4.2. Simulation Results and Discuss

The resource allocation algorithm based on bidding and two-tier DRL proposed in this paper is divided into two tiers.

**Figure 2:**
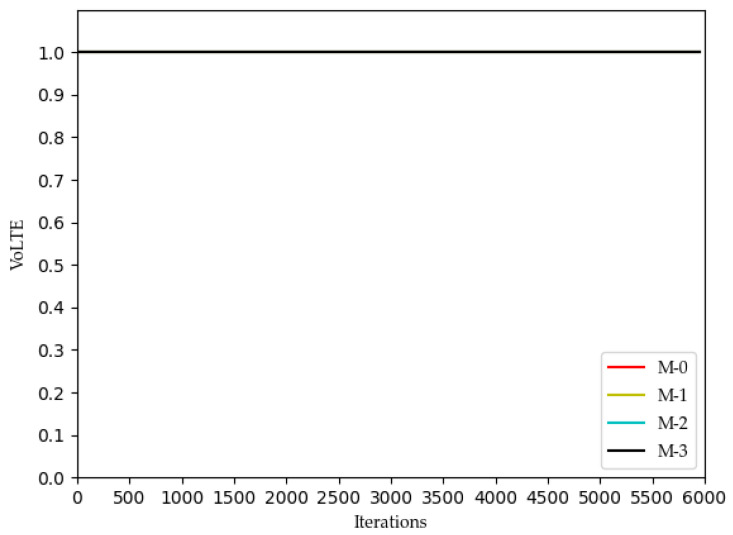
Optimization curve of QoE of VoLTE for each MVNO in the lower model based on DQN.

**Figure 3:**
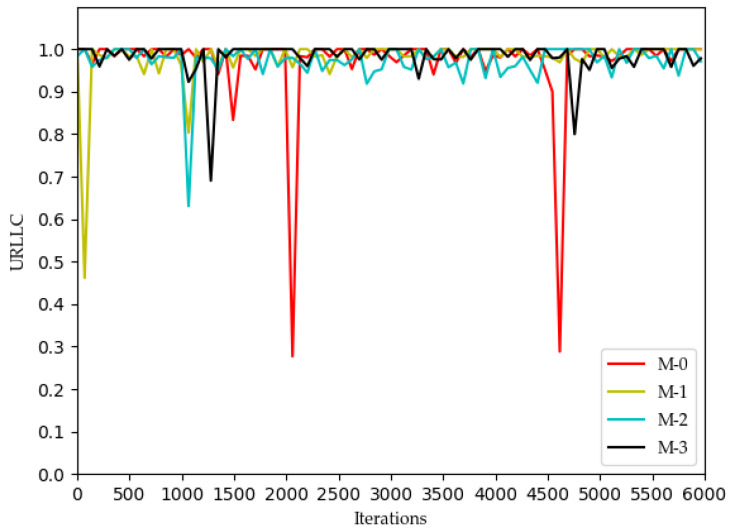
Optimization curve of QoE of URLLC for each MVNO in the lower model based on DQN.

**Figure 4:**
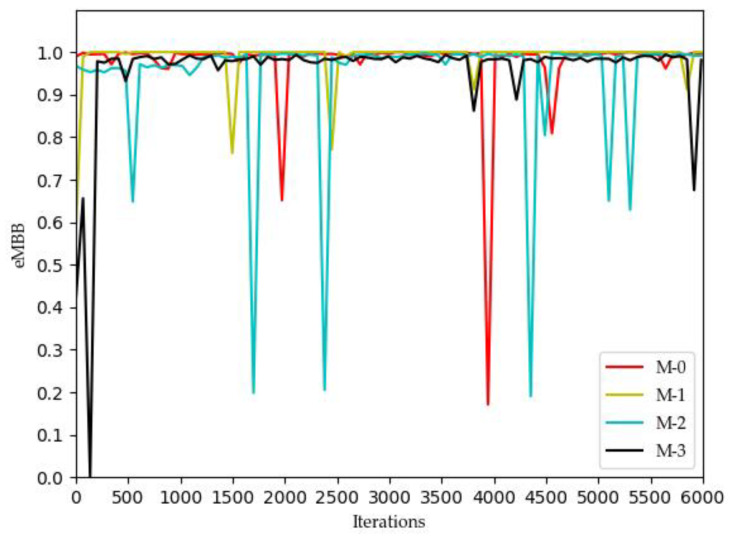
Optimization curve of QoE of eMBB for each MVNO in the lower model based on DQN.

**Figure 5:**
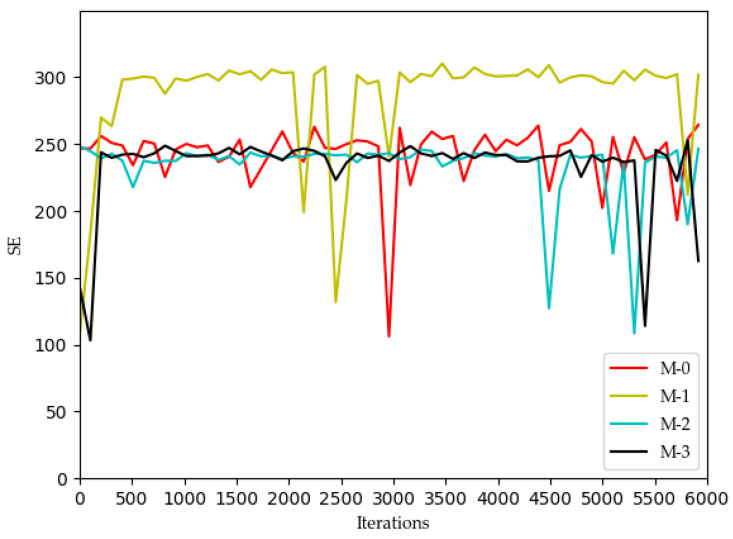
Optimization curve of SE for each MVNO in the lower model based on DQN.

Figure 2, Figure 3, Figure 4 and Figure 5 show the optimization curves of the QoE of three types of services and SE using the proposed algorithm in the lower tier.

We can see from Figure 2 that the QoE of VoLTE service reaches 1 without optimization, because the required rate requirement is very small (51 kbs). Providing a small part of the bandwidth for this service can meet its requirements. From Figure 3 and Figure 4, the QoE of URLLC and eMBB services fluctuate because the rate requirements of these two services are large (0.1 Gbs and 1 Gbs). Nevertheless, the QoE of these two services is maintained between 0.96 and 1.0. Some abnormal values in subsequent iterations are trial and error attempts made by dueling the DQN algorithm to prevent over optimization.

**Figure 6:**
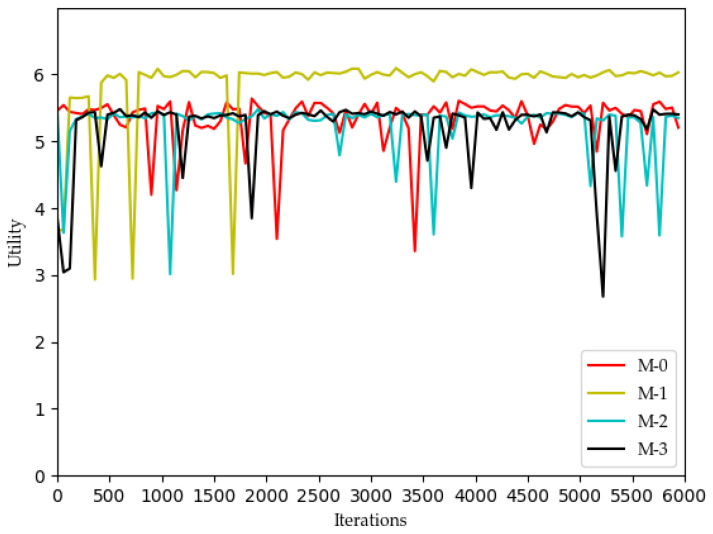
Optimization curve of SE for each MVNO in the lower model based on DQN.

It can be seen in Figure 5 and Figure 6 that the curves of the SE graph are significantly different from the curves of the QoE graphs of the other three services, and the SE curve has a strong correlation with the system utility curve compared to the three service curves.

However, for each MVNO, its system utility functions and SE shows significant optimization with increasing iterations, which confirms that using the Dueling DQN algorithm is a suitable choice for the model optimization problem proposed in this paper. In MVNO-1, for example, the SE curve fluctuates a lot before 400 iterations, and after 400 iterations, the SE curve has converged to the maximum value of 300 and tends to be stable, with a few low values after more than 400 iterations but does not affect the overall trend. The reason for this phenomenon is that the training neural net parameters were set to be replaced every 200 iterations during the simulation. The neural net parameters were in a relatively poor state when the training was first performed using DQN, and most of the assigned actions obtained from the initial neural net parameters and strategy selection were randomly selected actions in the action space, so the curve showed substantial fluctuations at the beginning. When the number of iterations reaches 400 and the neural net parameters in the Dueling DQN algorithm reach better, the subsequent choices of the allocations all appear to be better choices.

**Figure 7:**
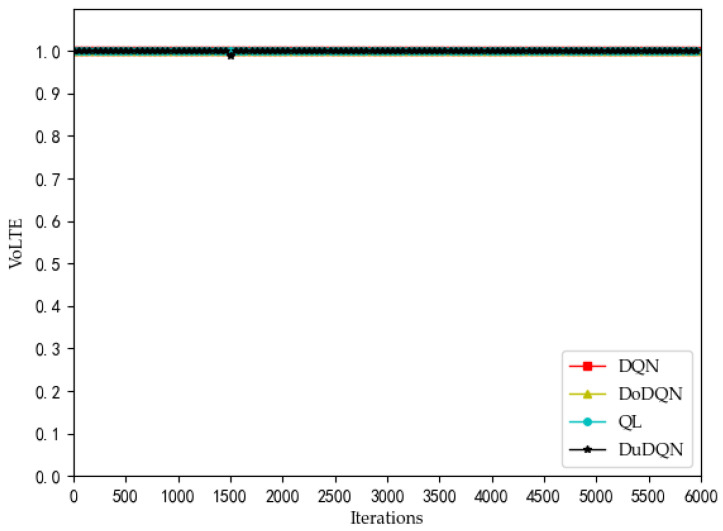
Comparison of QoE of VoLTE using different methods (taking MVNO-1 as examples).

**Figure 8:**
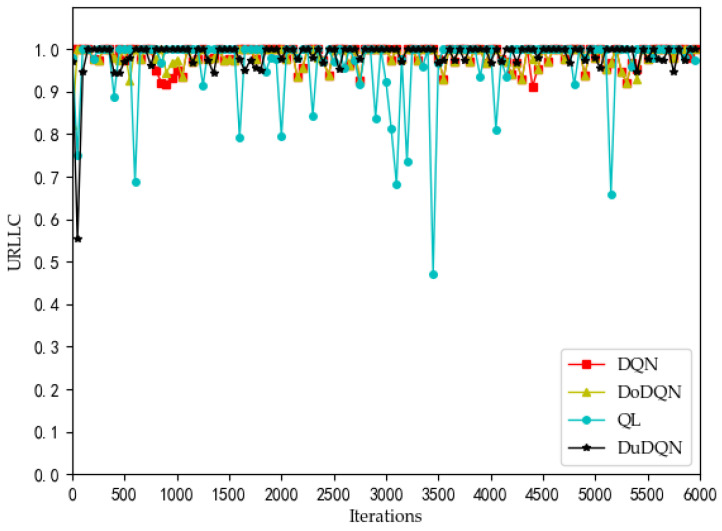
Comparison of QoE of URLLC using different methods (taking MVNO-1 as examples).

**Figure 9:**
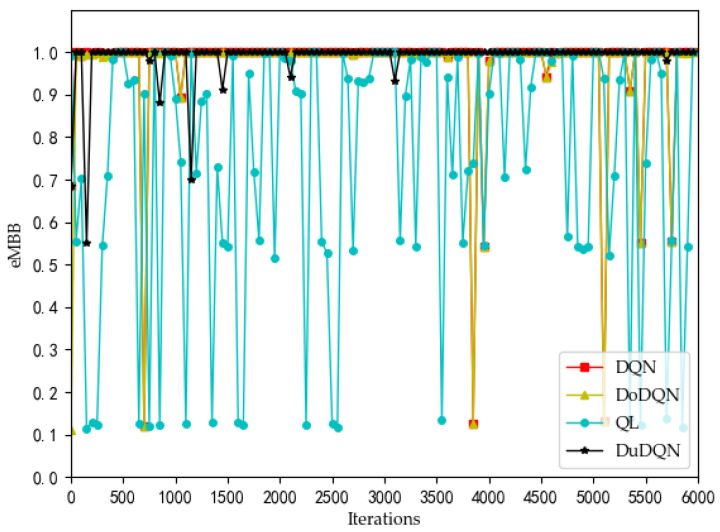
Comparison of QoE of eMBB using different methods (taking MVNO-1 as examples).

**Figure 10:**
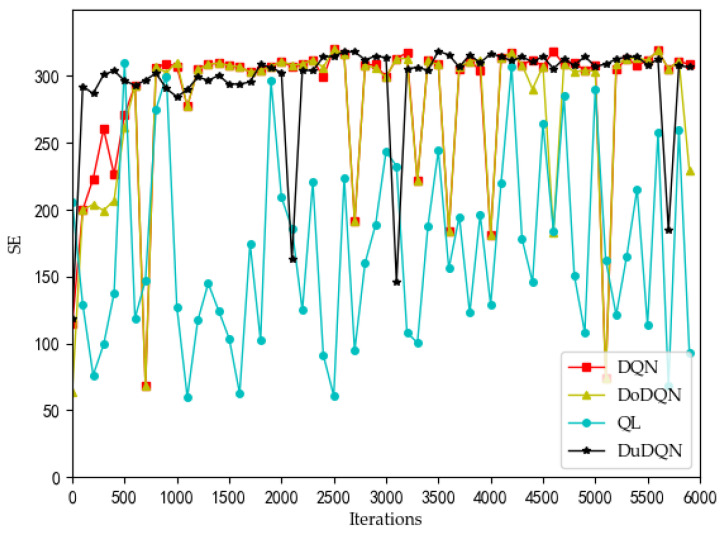
Comparison of SE using different methods (taking MVNO-1 as examples).

**Figure 11:**
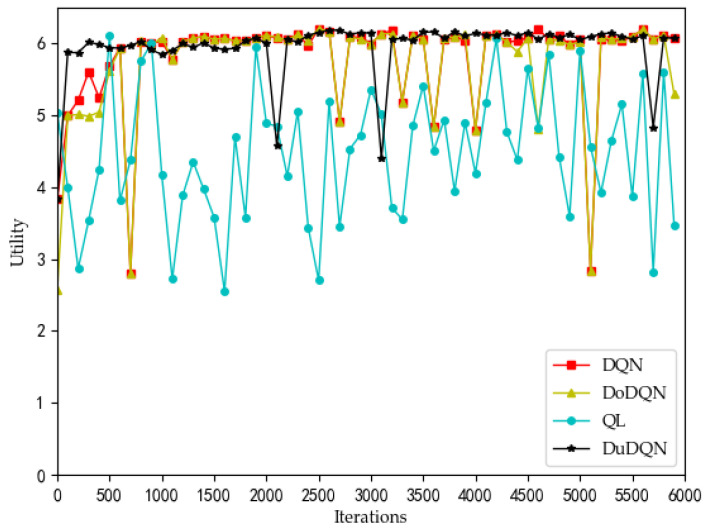
Comparison of utility using different methods (taking MVNO-1 as examples).

The changes in system utility, QoE, and SE for MVNO-1 with an increasing number of iterations using different methods are shown in Figure 7, Figure 8, Figure 9, Figure 10 and Figure 11.

Analyzing the curves of QoE for three service types (Figure 7, Figure 8 and Figure 9), it can be seen that for VoLTE service, the QoE values of all three methods are stable at 100%. For URLLC service and eMBB service, the QoE values of four algorithms show some fluctuations of low values, but all three methods are basically stable at 100%. However, the QoE curves of the three services obtained by the Dueling DQN algorithm are more stable and less volatile than the other three algorithms. It can be observed from the curves of system utility and SE (Figure 10 and Figure 11) that the DRL algorithms have a significant improvement over the QL algorithm.

For the curves of SE and system utility, the curves using the Dueling DQN algorithm have higher values than the curves of the other methods, and the curves converge and stabilize at the highest values (SE > 300, utility > 6). After 2200 iterations, the actual simulation data show that the SE obtained by the Dueling DQN algorithm is about 1% higher than that of the DQN algorithm, about 2.7% higher than the Double DQN algorithm, and about 76% higher than the QL algorithm.

And utility has also been slightly improved.

These four algorithms have obvious optimization for the whole system, and the SE and utility curves have obvious optimization trends. Through comparison, it is concluded that the curve obtained by the Dueling DQN algorithm is more stable than other centralized algorithms. Especially after 2200 iterations, the curve obtained by the Dueling DQN algorithm rarely fluctuates greatly, and even its average value converges to a relatively high value, which shows that using the Dueling DQN algorithm to solve the optimization problem of the lower model is a very effective method.

**Figure 12:**
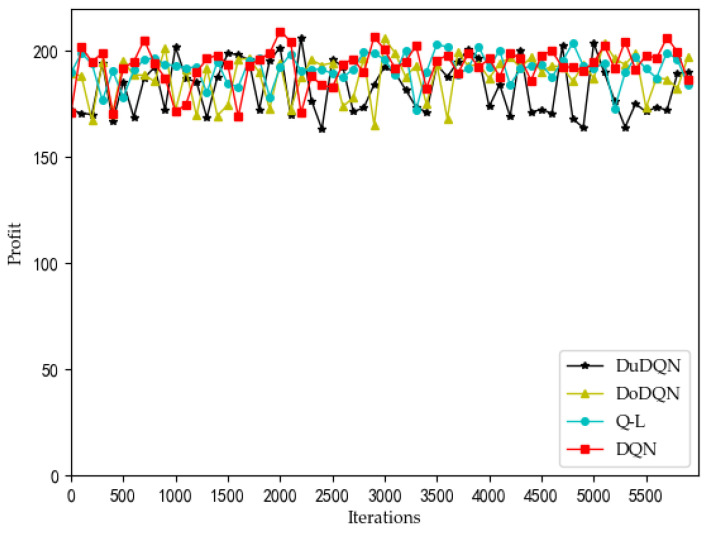
Comparison of profit using different methods.

**Figure 13:**
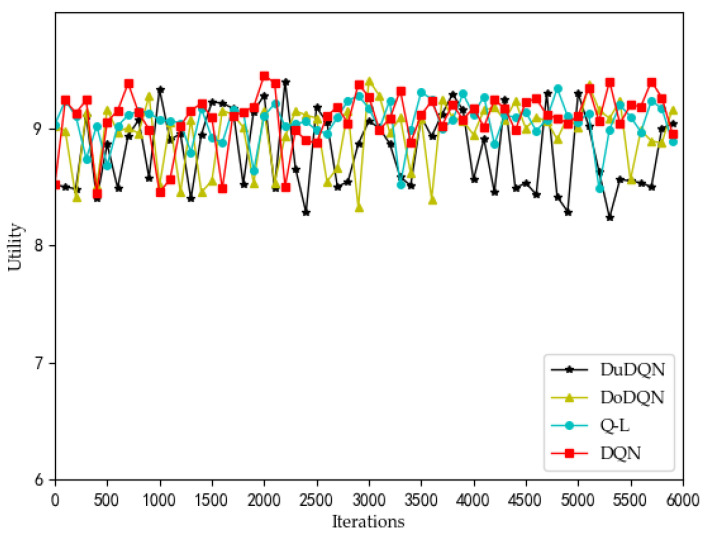
Comparison of utility using different methods.

Figure 12 and Figure 13 show the comparison of profit and utility of the upper model using different algorithms.

It can be seen from the line graph that the optimization effect of the upper model using the DQN algorithm (red curve) is the best. After the number of iterations reaches 3500, the profit of MVNO and the utility function of the system gradually converge to about 200 and 9, respectively. The second is the QL algorithm, whose curve is significantly higher than that of the other two algorithms, but after 3500 iterations, it is more volatile than that of the DQN algorithm. The two curves obtained by the Double DQN and Dueling DQN algorithm perform worse. As the advantages of the DQN algorithm over the other three algorithms cannot be clearly seen from the line graph, the violin graph is used to analyze and compare the data.

In the violin diagram, the wider the blue width is, the higher the ratio of the value here is. The middle line represents the mean value, and the upper and lower lines represent the maximum and minimum values.

**Figure 14:**
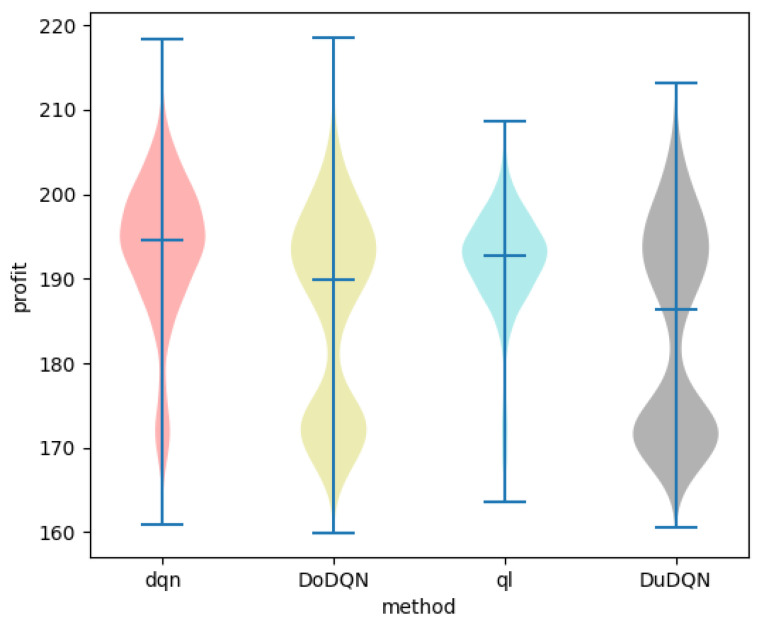
Comparison of profit using different methods.

**Figure 15:**
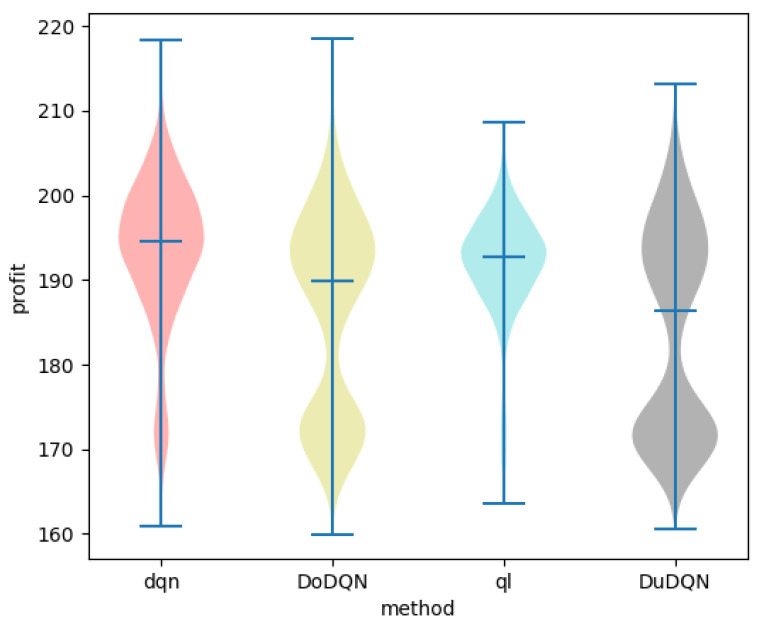
Comparison of utility using different methods.

It is obvious from Figure 14 and Figure 15 that, the system utility and MVNO benefit obtained by using the DQN algorithm are better than the other algorithms. The average values of Se and utility obtained by the DQN algorithm are the largest, and the values obtained are concentrated in a relatively high range, which is about 5.4% higher than Double DQN and about 2.6% higher than Dueling DQN. The reason may be that Double DQN and Dueling DQN improved by the DQN algorithm pay too much attention to the behavior of trial and error, but reduce the optimization effect of the system.

In general, it can be seen from Table 4, that the algorithm proposed in this paper is better than the comparison method in optimization performance, convergence speed, and convergence stability.

### 4.3. The Complexity Analysis

In terms of time complexity, the algorithm proposed in this paper needs to generate the state after the interaction between the environment and MVNO in each iteration, so it is difficult to obtain the operation time required by the algorithm in each iteration. However, the preset number of iterations in this paper is 6000.

From the perspective of spatial complexity, the spatial complexity of the DRL algorithm is obtained according to the number of neural network parameters, real-time addition Ca, and real-time multiplication cm that needs to be stored. The DRL algorithm used in this paper uses K hidden full connection layers, and each hidden layer is set with $o_{K}$ neural units.

(25) $$C_{P}=\sum_{k=1}^{K}(o_{k}+1)o_{k+1}$$

(26) $$C_{ℳ}=\sum_{k=1}^{K}o_{k}o_{k+1}$$

(27) $$C_{A}=\sum_{k=1}^{K}o_{k}o_{k+1}+\sum_{k=1}^{K}o_{k+1}$$

The neural network set up in this paper uses the Relu activation function, the number of hidden layers K=2, and the number of neurons in the two hidden layers $o_{K}$, Therefore, according to formula (22) and formula (25)–(27), we can get the spatial complexity:

(28) $$C=C_{ℒ}^{V}+C_{ℒ}^{A},\;\;\;\;ℒ\in \left\{P,\;ℳ,\;A\right\}$$

Therefore, it can be deduced that the complexity of the proposed algorithm is low. in addition, from the results, it can be seen that the proposed algorithm can converge at a faster speed and get the optimization results.

## 5. Conclusions

In this paper, we propose a two-tier slicing resource allocation algorithm with Dueling DQN and joint bidding to solve the optimization problem of resource allocation for multiple users in RAN scenarios. We first combine Dueling DQN and bidding in the upper tier of the proposed model to try to maximize the utilization of the BS resources, using an exhaustive enumeration to obtain the optimal lower tier actions corresponding to the upper tier actions, and using a penalty function to prevent the MVNOs from overbidding. The Dueling DQN is used in the lower tier of the model to allocate the resources to the users connected by each MVNO. Also, in this paper, bidding is combined with the Q-learning algorithm in the upper tier of the model, and the hard slicing approach is combined with bidding and used as a comparison to conclude that using the Dueling DQN algorithm in combination with bidding exhibits better performance. The use of the Dueling DQN algorithm in the lower tier also shows superiority over the use of the Double DQN algorithm, DQN algorithm, and the Q-Learning algorithm. In future work, it can take into consideration changes in user location and changes in service demand, in order to get closer to the actual communication scenario. And improve the proposed two-tier model by combining the bidding algorithm with more advanced DL algorithms to obtain a better allocation scheme.

## Figures and Tables

**Table 1:** Comparison with reference algorithm.

Reference	Two Tier Resource Allocation	Multiple Service Types	Network Slicing	DRL Joint Method
proposed algorithm	Yes	Yes	Yes	bidding
[3]	no	yes	yes	no
[12]	yes	no	no	no
[38]	no	yes	yes	BER
[39]	no	yes	yes	no

**Table 2:** Parameter settings for each service slice.

	VoLTE	eMBB	URLCC
System Bandwidth	10 MHZ		
Resource Block Specifications	0.2 MHZ		
BS Transmitting Power	46 dBm		
User Transmission Power	20 dBm		
Signal Path	Rayleigh decline		
Number of MVNOs	4		
Number of Users	Total: 100		
User Package Size	Constant: 40 Byte	Average value: 100 Byte, maximum value: 250 Byte	Constant: 0.3 MByte
SLA (speed):	51 kbs	100 Mbs	10 Mbs

**Table 3:** Number of users with different service requirements connected by MVNO.

	Number of Users with Different Service Needs		
	eMBB	VoLTE	URLLC
MVNO-0	11	9	7
MVNO-1	11	8	7
MVNO-2	8	6	13
MVNO-3	2	8	7

**Table 4:** The proposed algorithm is compared with other algorithms.

		QL	DQN	Dueling DQN	Double DQN
Upper tier	Profit	191 (1.5%)	194 (Proposed)	189 (2.6%)	184 (5.4%)
	Total Utility	9.01 (1.1%)	9.13 (Proposed)	8.91 (2.5%)	8.84 (3.2%)
Lower tier	Qoe of services	More unstable	More unstable	More unstable	More unstable
	SE	168 (76%)	294 (1%)	297 (Proposed)	289 (2.7%)
	Utility	4.43 (34.1%)	5.90 (0.5%)	5.93 (Proposed)	5.85 (1.3%)

## Data Availability

Data sharing not applicable.
